# Deterioration of Intended Target Volume Radiation Dose Due to Anatomical Changes in Patients with Head-and-Neck Cancer

**DOI:** 10.3390/cancers13174253

**Published:** 2021-08-24

**Authors:** Olga Hamming-Vrieze, Simon van Kranen, Iris Walraven, Arash Navran, Abrahim Al-Mamgani, Margot Tesselaar, Michiel van den Brekel, Jan-Jakob Sonke

**Affiliations:** 1 Department of Radiation Oncology, Netherlands Cancer Institute, 1066 CX Amsterdam, The Netherlands; s.v.kranen@nki.nl (S.v.K.); i.walraven@nki.nl (I.W.); a.navran@nki.nl (A.N.); a.almamgani@nki.nl (A.A.-M.); j.sonke@nki.nl (J.-J.S.); 2 Department of Medical Oncology, Netherlands Cancer Institute, 1066 CX Amsterdam, The Netherlands; m.tesselaar@nki.nl; 3 Department of Head and Neck Surgery, Netherlands Cancer Institute, 1066 CX Amsterdam, The Netherlands; m.vd.brekel@nki.nl

**Keywords:** head-and-neck cancer, cone beam CT, dose accumulation, adaptive radiotherapy

## Abstract

**Simple Summary:**

Delivered radiation dose in the patient during a course of 6–7 weeks of treatment can differ from intended radiation dose in the treatment planning. This study analyzes these dose differences in the target volumes in a set of 188 head-and-neck cancer patients. It was found that large dose deteriorations in targets occur in a minority of patients, although more frequently when smaller margins were used. The correlation to visual estimation of differences based on changing anatomy was poor. Therefore, dosimetric selection tools during treatment to assess differences seem warranted to identify patients at risk for under or overdosage. With such tools, patients at risk can be selected to adjust the treatment plan during treatment (adaptive radiotherapy) to correct the radiation dose.

**Abstract:**

Delivered radiation dose can differ from intended dose. This study quantifies dose deterioration in targets, identifies predictive factors, and compares dosimetric to clinical patient selection for adaptive radiotherapy in head-and-neck cancer patients. One hundred and eighty-eight consecutive head-and-neck cancer patients treated up to 70 Gy were analyzed. Daily delivered dose was calculated, accumulated, and compared to the planned dose. Cutoff values (1 Gy/2 Gy) were used to assess plan deterioration in the highest/lowest dose percentile (D_1_/D_99_). Differences in clinical factors between patients with/without dosimetric deterioration were statistically tested. Dosimetric deterioration was evaluated in clinically selected patients for adaptive radiotherapy with CBCT. Respectively, 16% and 4% of patients had deterioration over 1 Gy in D_99_ and D_1_ in any of the targets, this was 5% (D_99_) and 2% (D_1_) over 2 Gy. Factors associated with deterioration of D_99_ were higher baseline weight/BMI, weight gain early in treatment, and smaller PTV margins. The sensitivity of visual patient selection with CBCT was 22% for detection of dosimetric changes over 1 Gy. Large dose deteriorations in targets occur in a minority of patients. Clinical prediction based on patient characteristics or CBCT is challenging and dosimetric selection tools seem warranted to identify patients in need for ART, especially in treatment with small PTV margins.

## 1. Introduction

Radiation therapy is state of the art in the organ preserving treatment of head-and-neck cancer. With modern radiotherapy planning techniques, steep dose gradients can be created around target volumes. However, during treatment, anatomical changes could negatively affect these planned dose distributions, examples are weight loss or change in tumor size and location [1]. Several studies investigated differences between planned and actual delivered doses to target volumes calculated on repeat imaging. The reported results are contradicting, where some studies reported target volume underdoses [2,3,4,5,6,7], other studies reported no differences [8,9,10] and a few reported target volume overdoses [5,11,12].

To counteract dose deviation during treatment and improve accuracy, adaptive radiotherapy has been developed (ART). With ART, the radiation treatment plan is adjusted during the course of radiation. Clear procedures to identify patients who require ART during treatment are not available. In clinical practice, patients are typically selected at the discretion of the physician, often considering gross anatomical deformations visualized with in-room imaging. Thus, patient selection is subjective and it is unclear if the current practice to select patients at risk of dose deterioration is effective.

Moreover, ART is labor intensive. Frequently, new imaging is acquired to re-plan the radiation treatment. Alternatively, adjustment of delineated volumes and re-planning is performed on the original planning CT. The balance between the availability of resources and benefit of ART would be improved with accurate patient selection of those in need to correct dose deterioration.

The aim of this work was to quantify dose deterioration in the target volume by comparing planned versus delivered dose (without adaptive intervention), to identify predictive factors for the likelihood of dose deterioration, and to compare dosimetric results to clinically selected patients based on gross anatomical changes on CBCT.

## 2. Materials and Methods

One hundred and eighty-eight consecutive head-and-neck cancer patients, treated from 2013 to 2016 with a simultaneous integrated boost radiotherapy plan to a total dose of 70 Gy, were selected. Institutional approval was acquired for this analysis. Details of patient positioning and planning CT (pCT), high and low dose clinical target volume delineation (CTV1 and CTV2, respectively), dose prescription, and planning objectives are given in supplemental material Table S1. A planning target volume (PTV) was constructed around all CTVs, 5 mm margin was used in patients treated before 1 April 2015 (90 patients), thereafter, a PTV margin of 3 mm was applied (98 patients). Treatment plans were optimized in Pinnacle (Philips Medical Systems, Eindhoven, The Netherlands). During treatment, daily CBCT scans (Elekta Synergy, Elekta Oncology systems, Crawley, UK) with 1 mm^3^ voxel size were acquired. CBCT scans were used for online positioning by alignment of bony anatomy close to the CTVs [13].

**Figure 1:**
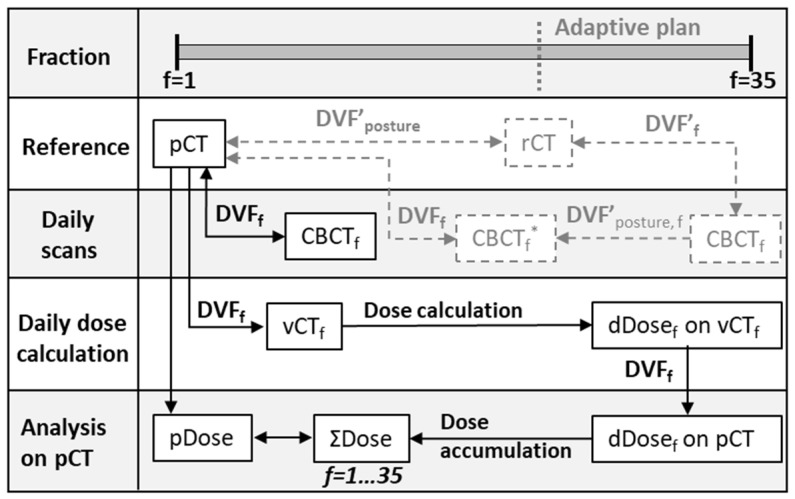
Schematic overview of workflow to calculate the daily delivered dose.

Daily CBCT anatomy was used to calculate the daily delivered dose (Figure 1). First, each CBCT was shifted to account for daily couch correction. Then, a deformable image registration with a B-spline deformation model was used to deform the pCT to the anatomy of the day in the CBCT [14,15]. With the derived deformation vector field, a virtual CT with the correct Hounsfield units (vCT_f_) was obtained for daily dose recalculation. In the event of a missing CBCT, e.g., due to maintenance, the daily dose was estimated based on fractions surrounding the missing CBCT. For courses with adaptive interventions, we simulated the accumulated dose as if the initial plan had been delivered for all fractions. A correction was applied to account for posture changes due to a new mask. These changes were captured by comparing the repeat CT with the pCT and propagated into the daily CBCT. Calculated daily doses were mapped back to the pCT for accumulation.

For comparison of planned doses with accumulated doses, evaluation target volumes (ETVs) in between the CTV and PTV were created by expanding CTVs with 2 mm. On the one hand, this was done to include geometrical uncertainties such as delineation errors and registration inaccuracies, which are overlooked when evaluating on CTV only. On the other hand, to exclude residual setup and inter-fraction anatomical changes, which are already explicitly accounted for in the dose accumulation.

Planned doses on PTVs were compared to the accumulated dose on ETVs (without adaptive intervention). Cutoff values of 1 and 2 Gy (corresponding to the fraction size (half or complete) were used to count the number of patients with plan deterioration in high and low dose target volumes (TV1 and TV2). Overdose was evaluated in D_1_ (1% of the volume receives this dose value or higher), underdose in D_99_ (the minimum dose to 99% of the hottest part of the volume). To evaluate the number of patients with dose deterioration in any of the target volumes, incidence of the worst case absolute dose deterioration was plotted, both for over- and under-dose. Predictive factors per target volume and endpoint were statistically evaluated. Furthermore, dosimetric results were compared to clinically selected patients for whom ART was performed during treatment based on visualized gross changes on CBCT with concern for over- or under-dosage of target volumes, for instance, due to decrease in neck diameter or progression/shift of target volumes. For reference (in comparison to the analyses of planned PTV to accumulated ETV), the percentage of patients with plan deterioration comparing PTV to CTV, CTV to CTV, and PTV to PTV were stated.

### Statistical Analysis

Baseline characteristics are presented as the percentage for categorical variables or mean (±SD) for continuous variables (Table 1). Differences between patients with and without dosimetric deterioration were tested using the independent samples *t*-test for normal distributed continuos variables and Chi-Square-tests for categorical data or a Fisher’s exact-test in case of less than five events in at least one category. The analyzed factors were age, gender, baseline weight and BMI, weight change during treatment, tube feeding during treatment, tumor pathology, tumor location, tumor stage, nodal stage, uni or bilateral nodal stage, largest nodal size, radiation scheme (Dahanca (35 fractions in 6 weeks) versus conventional (35 fractions in 7 weeks) radiation scheme), concurrent chemotherapy (yes versus no), and PTV margin (5 verus 3 mm). Significant factors on these tests were further analyzed with binary logistic regression to investigate the predictive performance of baseline variables on dose deterioration. First, linearity of the association between continuous independent variables and the outcome variable was tested and in case of a non-linear association, the variable was grouped into quartiles. Second, univariable logistic regression analyses were performed to identify predictive single parameter models for dose deterioration per evaluated target volume. Third, the predictive accuracy of these models were estimated using the discriminatory ability of the models by calculating the area under the receiver operating characteristic curves (AUC). Discrimination is the ability to distinguish between those with dose deterioration during treatment from those without. The discriminatory ability is graded as poor for an AUC below 0.7, moderate between 0.7 and 0.8, good between 0.8 and 0.9, and excellent if >0.9 [16]. A two-sided *p*-value of <0.05 was considered significant. Multivariable analyses was not performed due to the low number of events. IBM SPSS Statistics version 24 was used.

Daily cone beam CT (CBCT) anatomy was used to estimate the actual delivered dose. Deformable image registration was used to calculate a deformation vector field (DVF_f_) to map the planning CT (pCT) to the CBCT for each fraction f. The resulting daily virtual CT (vCT_f_) was used to calculate the daily delivered dose. For CBCTs acquired after adaptive intervention, a correction was applied to account for posture changes due to a new mask. These changes were captured by comparing the repeat CT (rCT) with the pCT and propagated (DVF’_posture,f_) into a CBCT *. The daily dose (dDose_f_) was subsequently mapped back to the pCT for accumulation (Σdose) and comparison to the planned dose (pDose).

## 3. Results

**Figure 2:**
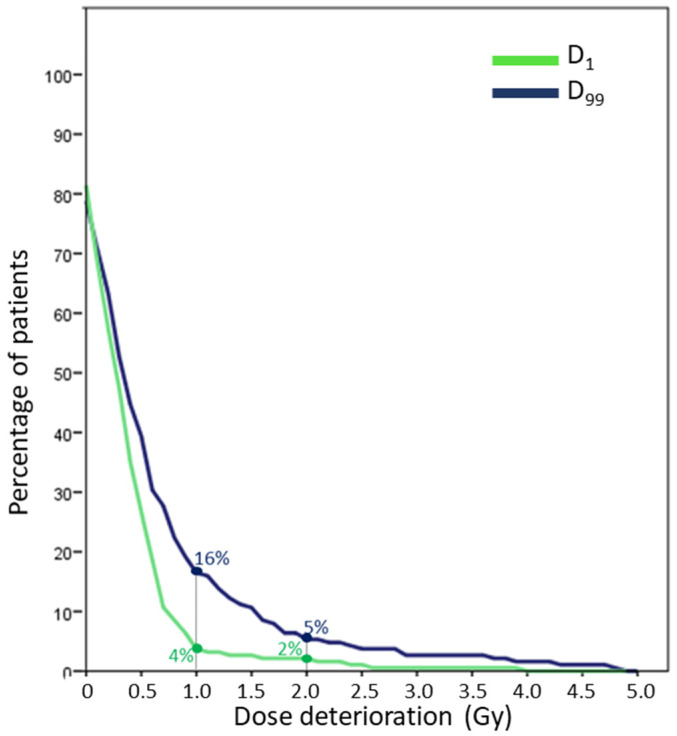
Incidence of the worst case absolute dose deterioration between accumulated ETV and planned PTV in any of the target volumes, both for over- and under-dose (D_1_ and D_99_, respectively).

The incidence of absolute dose deterioration comparing accumulated ETV with planned PTV doses in any of the target volumes is plotted in Figure 2. Sixteen and 4% of patients had deterioration over 1 Gy in D_99_ and D_1_, respectively which was 5% (D_99_) and 2% (D_1_) over 2 Gy.

An overview of the analyzed factors is given in the supplemental material Tables S3a–d and S4. Significant logistic regression results of dose deterioration over 1 Gy only are stated in the following paragraphs considering the low number of events in dose deterioration over 2 Gy (Table S2).

### 3.1. Minimum Dose (D_99_)

Analyzing both target volumes separately showed that underdosage over 1 Gy in TV1 and TV2 was present in 8% and 13% of patients, respectively, and in 2% and 4% of patients over 2 Gy.

**Figure 3:**
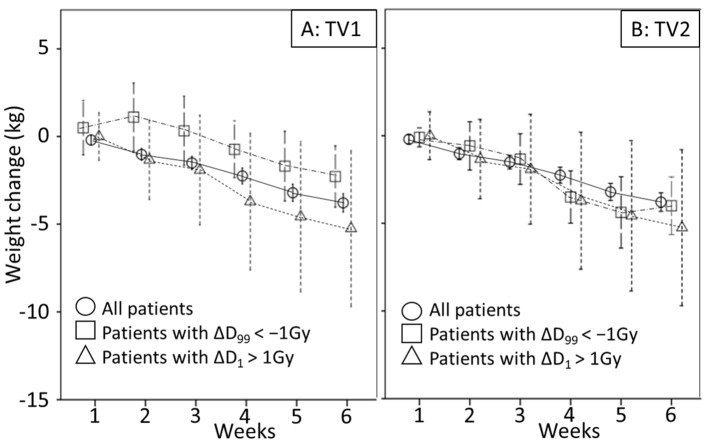
Mean weight loss comparing all patients to patients with dose deterioration are shown in (**A**) for target volume 1 (TV1) and in (**B**) for target volume 2 (TV2).

Factors significantly associated with deterioration of D_99_ in TV1 were weight gain in the second and in the third week of treatment (Table 2, Figure 3). Weight change in week 2 was a stronger predictive factor than in week 3 and had an AUC of 0.74. Furthermore, all deteriorations were observed in the 3 mm PTV margin group (Table 2 and Table S3a).

Risk factors for deterioration of D_99_ over 1 Gy in TV2 were higher baseline weight and BMI (Table 2). Furthermore, deteriorations were predominantly observed in the 3 mm PTV group (Table 2 and Table S3b). PTV margin was the strongest predictive factor with an AUC of 0.70.

### 3.2. Maximum Dose (D_1_)

Overdosage >1 Gy in TV1 and TV2 was present in 4% of cases in both volumes, >2 Gy was present in 2% and 1% of patients, respectively. For both target volumes, the same two factors were significantly associated with an increased risk of plan deterioration with D_1_ > 1 Gy: Higher baseline weight and higher T stage (Table 2). Baseline weight was a stronger predictor than a higher T stage for both target volumes (AUC 0.74, Table 2). PTV margin was not associated with an increased risk of D_1_ dose deterioration (Table S3c,d).

### 3.3. Clinical Selected Patients for ART

Of the 188 patients, 24 patients received an adapted treatment plan due to the anatomical changes with visual estimation of target dose deterioration, either under or overdosage of the CTVs. Table 3 presents a confusion matrix between clinical patient selection with CBCT to dosimetric deviations. The sensitivity (the proportion of patients clinically selected for ART who have a dose deterioration) was 22% for dosimetric changes larger than 1 Gy and 29% for changes larger than 2 Gy, the specificity (the proportion of patients without gross CBCT changes who did not have dosimetric changes) was 89% both for dosimetric changes larger than 1 and 2 Gy. Correlation of clinical estimation of dose deterioration with dosimetric changes was strongest for underdosage of the high dose target volume (Table S5).

### 3.4. Reference and Evaluated Volumes

**Figure 4:**
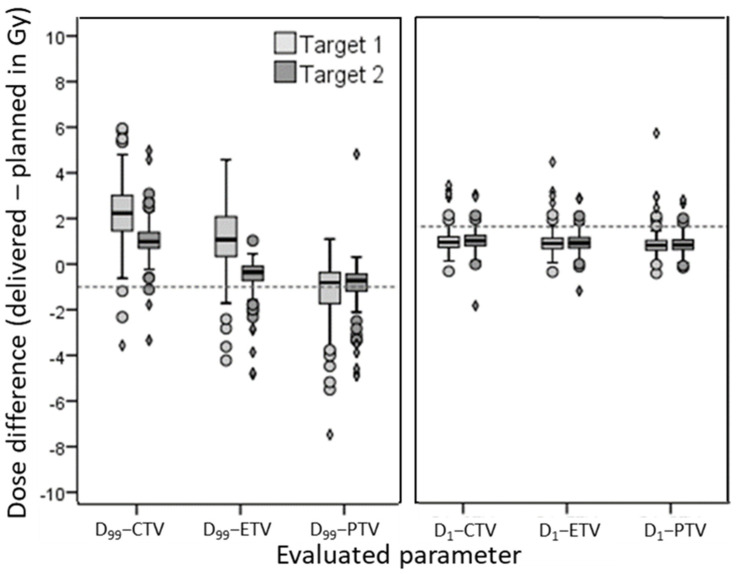
Demonstrates the implication of comparing different volumes. This figure shows boxplots of the change between planned PTV dose versus accumulated dose in the PTV, ETV, and CTV for D_1_ and D_99_, both for target 1 (high dose volume) and 2 (low dose volume). A cutoff line is drawn at minus 1 Gy for D_99_ and plus 1 Gy for D_1_.

D_99_ and D_1_ dose differences comparing planned PTV dose to accumulated dose in either CTV, ETV or PTV, are depicted in Figure 4. Results of threshold methods using D_99_ dose deterioration depend on the distance between planned and evaluated volume, an increase in distance resulted in the decrease of threshold violation. In contrast, D_1_ dose differences were rather similar for all evaluated volumes. For reference, the percentage of patients with plan deterioration beyond the 1 and 2 Gy threshold, comparing PTV to ETV, PTV to CTV, CTV to CTV, and PTV to PTV, are stated in Table S2.

## 4. Discussion

In this paper, deterioration of the intended target volume dose was evaluated by estimating the actual delivered dose. Large dose deteriorations in target volumes occurred in a minority of patients. Performance of predictive models were moderate at best depending on the evaluated target volume and dose parameters. Furthermore, patient selection based on gross anatomical changes visualized on CBCT showed a low sensitivity for detection of calculated dosimetric differences.

As mentioned in the introduction, studies evaluating differences between planned and delivered doses in target volumes are contradictory [2,3,4,5,6,7,8,9,10,11,12]. Multiple factors may contribute to these contradicting results. For example, the limited number of patients that were included in these studies (10–37 patients), the definition and location of target volumes, a variability in planning techniques and proximity of dose gradients, the use of different dose accumulation methods and time points used, the choice in compared volumes (i.e., PTV to CTV or CTV to CTV) and DVH parameters (i.e., D_95_, D_98_, D_99_, mean dose, median dose, V_107_). Moreover, it is not clear which criterion to use to define dose differences. In this work, deterioration in dose was set out to a range of thresholds. When applying thresholds corresponding to the fraction size (half or complete), we found that the planned D_99_ and D_1_ deteriorated in 16% and 4% of patients, respectively more than 1 Gy, this percentage dropped to 5% and 2%, respectively using a 2 Gy threshold. Appropriate thresholds are likely to be endpoint specific since consequences differ between overdosage and underdosage. The minimum dose, for instance D_99_, is a surrogate for tumor control probability. Ideally, tumor control probability models should be used to predict the clinical effect of dose deterioration. In the absence of such models, the absolute dose deterioration or deterioration below a certain cutoff (i.e., 95% of prescribed dose) could be used. Overdosage can result in increase of toxicity, especially the occurrence of late mucosal ulcers. Olteanu et al. [17] analyzed predictive factors and advocated using a dosimetric threshold for the peak-dose volume, for instance, the volume receiving 84 Gy or more.

The ability of ART to restore the dose distribution with a re-plan and enhance clinical outcome still remains to be determined. Planning comparative studies show that ART has the ability to improve the dose distribution, although not every patient will benefit [2,4,7]. Castelli et al. reported on an in silico study with 37 head-and-neck-cancer patients [4]. Comparison of delivered dose without ART to weekly ART showed an increase of target coverage in a majority of patients, the median D_98__CTV increased from 68 to 69.2 Gy. The available evidence of the clinical benefit with improved tumor control from ART is sparse. The largest prospective trial comparing radiotherapy with ART to the standard treatment was performed in nasopharyngeal carcinoma patients in China [18]. In this trial, patients were not randomized, but could choose to receive ART. This non-randomized design introduced confounders, for instance, fitter patients are more likely to have chosen the ART group, thereby introducing a bias. The study showed a significantly better 2 year locoregional control in the ART group (97% in 86 ART-patients versus 92% in 43 no-ART-patients). Moreover, a large retrospective study of head-and-neck carcinoma reported on by Chen et al. [19] showed an advantage of performing ART. Clinical outcomes of 51 ART-patients were compared to 266 no-ART-patients, results showed a 2 year locoregional control of 88% for ART-patients compared to 79% for no-ART-patients (*p* = 0.01). Furthermore, two smaller prospective single arm trials reported promising outcomes of patients treated with ART [20,21].

However, in determining the value of ART, we need to keep in mind that not all improvements are clinically relevant and patient selection is needed to let the benefit outweigh the effort of implementing ART [22]. In our study, factors associated with increased risk of dose deterioration were diverse and depended on the evaluated target volume and dose parameters. Single strong predictive models were not identified, but notable was the increased risk of underdosage with the use of smaller margins, both in low and high dose target volumes. In addition to the lack of strong predictive factors, other aspects add to the complexity of predicting which patient could benefit from ART. Even for evaluation of target volumes only, multiple endpoints can be defined, all with their own associated factors. Addition of predictive factors associated with dose deterioration in OAR will increase complexity. In combination with the relative low number of patients with dose deterioration, multivariable analyses or the development of a multifactorial prediction model is only possible in a very large series of patients. Alternatively, patient selection should focus on changes during treatment. However, the value of visualized anatomical changes on CBCT to select the patient for ART was limited in our cohort. Similar findings were published by Vickress et al. [23], they reported a series of 18 clinically selected patients for ART in which only seven needed adaptation based on dosimetrical findings. Therefore, dosimetric selection tools during treatment seem warranted, for instance, with regular dose recalculation to estimate dose deterioration at the end of treatment, both for target volumes, as well as OAR.

Although dose recalculation strategies are attractive, several issues should be addressed to optimize such a patient selection tool for ART: (1) Daily dose accumulation should be accurate. A key factor in dose accumulation is the use of deformable image registration (DIR) to take anatomical changes into account. Several registration algorithms are available, however, there is currently no consensus how to assess accuracy of resulting deformation vector fields. In a study using anatomical landmarks and implanted tumor markers to evaluate accuracy of B-Spline DIR, a precision of 1.8 mm for normal tissue and 3.3 mm for tumor tissue was found [24]. Additional uncertainties are introduced by complex non-elastic tumor response and (dis)appearing tissue or objects, for instance, tumor shrinkage or cavity filling. Biomechanical models, taking relational and intensity data into account, are proposed to mitigate the effect of changing mass [25,26]. (2) Prediction, preferably early during treatment, of the expected delivered dose at the end of treatment should be reliable. False positive patient selection could result in unnecessary re-planning and additional workload, while false negative patients might have decreased tumor control probabilities. Reliable prediction in the early phase of treatment will result in enough remaining fractions to correct dose deviations. It would also be beneficial for the decision process to evaluate a possible improvement with an ART step when evaluating predicted dose deviations. (3) Guidelines for thresholds to use for ART patient selection should be developed. As discussed above, currently, how accumulated dose should be evaluated and thresholds defined still remains unclear. Decision making in ART would greatly benefit if reliable TCP models were developed. In contrast, NTCP models are already widely available in literature, however such models harbor uncertainty and model validation is frequently lacking. In the Netherlands, the Dutch proton therapy platform is pursuing validation of NTCP models to use for model-based patient selection, thereby increasing the number of applicable NTCP models and creating a nation-wide consensus on their use [27]. Improving standardization of chosen parameters, used thresholds and compared volumes will result in better comparable research to analyze which patients clinically benefit from ART. (4) Technical issues such as speed, automation, archiving, and reporting should be optimized. ART procedures should be fast and mostly automated to limit clinical workload and allow a more generous patient selection. Nowadays, DIR algorithms, contour propagation, and dose recalculation engines are generally fast. However, automatic re-contouring harbors uncertainty and visual check of new contours is desirable, especially of areas without clear anatomical borders. Current ART strategies for head-and-neck cancer use offline re-planning of selected patients. Re-planning may be facilitated by starting a new plan optimization with the objectives and beam parameters from the original plan, although manual tweaking will most likely improve plan quality. Standardization of archiving and reporting will ensure patient safety and facilitate BIG data analyses for correlating image-dose-response data in a clinical utilizable manner [28].

The above mentioned issues also contribute to the limitations in our study such as the uncertainty incorporated in dose calculation, accumulation, and evaluation tools. Moreover, we demonstrated that comparing different volumes to each other can result in different percentages of patients marked with plan deterioration (supplemental material). Since the PTV is designed as a planning tool to achieve the desired dose distribution in the CTV, we argue that planned PTV doses should be compared to actual delivered CTV doses with an additional margin for errors not corrected for in the dose accumulation method. We chose an additional margin of 2 mm, however, this is not evidence based and a different expansion could influence results.

## 5. Conclusions

In conclusion, this study shows that most patients do not demonstrate significant dose deterioration, but with smaller margins the risk of dose deterioration increases. The use of predictive risk factors to select patients for ART is limited. Furthermore, patient selection based on gross anatomical changes visualized on CBCT does not appear to correlate well with the calculated dosimetric differences. Dosimetric patient selection tools, preferably automated, seem warranted to monitor possible dose deterioration and identify patients in need for re-plan.

## Figures and Tables

**Table 1:** Patient characteristics.

Patient and Treatment Characteristics (*n* = 188)	
Age (years, (SD))	61.1 (10.8)
Gender (*n*, (%))	
Male	133 (71)
Female	55 (29)
Weight (kg, (SD))	76.3 (17.9)
BMI at start (kg/m^2^, (SD))	24.9 (5.5)
Location (*n*, (%))	
Nasopharynx	31 (16)
Oroph/oral cavity	86 (46)
Larynx/Hypoph	43 (23)
Other	28 (15)
Pathology (*n*, (%))	
Squamous cell	174 (93)
Other	14 (7)
HPV	
No	64 (34)
Yes	47 (25)
Unknown	77 (41)
T stage (*n*, (%)) *	
Tx/T1/T2	109 (58)
T3/T4	79 (42)
N stage (*n*, (%)) *	
N0	66 (35)
N1-3	122 (65)
N side (*n*, (%))	
Unilateral	66 (54)
Bilateral	56 (46)
N largest size (cm, (SD))	2.3 (1.6)
N largest size (*n*, (%))	
<3 cm	82 (67)
≥3 cm	40 (33)
Dahanca (*n*, (%))	
No	94 (50)
Yes	94 (50)
Concurrent chemotherapy (*n*, (%))	
No	96 (51)
Yes	92 (49)
PTV margin (*n*, (%))	
5 mm	90 (48)
3 mm	98 (52)
Tube feeding (*n*, (%))	
No	124 (66)
Yes	64 (34)
Clinical ART (*n*, (%))	
No	164 (87)
Yes	24 (13)

SD: Standard deviation; *n*: Number; BMI: Body mass index; HPV: Human papilloma virus; PTV: Planning target volume; ART: Adaptive radiotherapy. * TNM 7th edition.

**Table 2:** Binary logistic regression analyses to identify significant factors for dose deterioration > 1 Gy.

Evaluated	Significant	Category	OR	95% CI	*p*	AUC	95% CI
D_99__TV1 (15 out of 188 had a threshold violation)	Weight ∆ week 2	<−2 kg	0.72	0.06–8.36	0.796		
		−2 to −1 kg	0.72	0.10–5.35	0.752		
		−1 to 0 kg	1.00				
		>0 kg	6.46	1.27–32.81	0.024	0.74	0.58–0.89
	Weight ∆ week 3	<−3 kg	0.73	0.06–8.34	0.800		
		−3 to −1 kg	3.40	0.62–18.30	0.158		
		−1 to 0 kg	1.00				
		>0 kg	5.40	1.02–28.44	0.047	0.70	0.56–0.84
	Weight ∆ week 4	<−4 kg	1.06	0.14–7.90	0.958		
		−4 to −2 kg	1.54	0.24–9.75	0.646		
		−2 to 0 kg	1.00				
		>0 kg	3.40	0.68–16.87	0.138		
	PTV margin	3 mm	n.a. *				
		5 mm					
D_99__TV2 (23 out of 177 had a threshold violation)	Weight	Per kg	1.03	1.01–1.06	0.005	0.67	0.55–0.79
	BMI	Per kg/m^2^	1.10	1.02–1.18	0.010	0.64	0.52–0.76
	Weight ∆ week 4	<−4 kg	1.50	0.43–5.22	0.530		
		−4 to −2 kg	1.40	0.40–4.88	0.597		
		−2 to 0 kg	1.00				
		>0 kg	0.41	0.09–1.83	0.242		
	PTV margin	3 mm	7.40	2.11–25.92	0.002	0.70	0.60–0.80
		5 mm	1.00				
D_1__TV1 (7 out of 188 had a threshold violation)	Age	Per year	1.06	0.98–1.14	0.144		
	Weight	Per kg	1.04	1.00–1.08	0.037	0.74	0.59–0.89
	BMI	Per kg/m^2^	1.11	0.99–1.24	0.056		
	T-category	T1/2	1.00				
		T3/4	8.88	1.05–75.28	0.045	0.73	0.56–0.89
D_1__TV2 (7 out of 177 had a threshold violation)	Age	Per year	1.06	0.98–1.14	0.132		
	Weight	Per kg	1.04	1.00–1.08	0.040	0.74	0.59–0.89
	BMI	Per kg/m^2^	1.11	0.99–1.24	0.056		
	T-category	T1/2	1.00				
		T3/4	8.78	1.03–74.57	0.046	0.73	0.56–0.89

* n.a.: not applicable.

**Table 3:** Confusion matrix of clinical selected patients for ART with dosimetric findings.

Threshold 1 Gy	Dosimetrical Yes	Dosimetrical No	Total
Clinical Yes	8 (4%)	16 (9%)	24 (13%)
Clinical No	28 (15%)	136 (72%)	164 (87%)
Total	36 (19%)	152 (81%)	188 (100%)
Threshold 2 Gy			
Clinical Yes	4 (2%)	20 (11%)	24 (13%)
Clinical No	10 (5%)	154 (82%)	164 (87%)
Total	14 (7%)	174 (93%)	188 (100%)

## Data Availability

The data presented in this study are available on request from the corresponding author.

## Supplementary Materials

The following are available online at https://www.mdpi.com/article/10.3390/cancers13174253/s1. Table S1: Details of patient positioning and planning CT, high and low dose clinical target volume delineation, dose prescription, and planning objectives; Table S2: Threshold violation of 1 and 2 Gy difference of accumulated versus planned dose; Table S3a–d: Association of characteristics with deterioration over 1 and 2 Gy of the accumulated dose versus the planned dose for D_1_/D_99_ in target volume 1 and 2; Table S4: *p*-values of the independent sample *t*-test evaluating differences between patients with and without dosimetric deterioration testing weight change and tube feeding during treatment; Table S5: Association of clinical ART with dosimetric deterioration over 1 and 2 Gy of accumulated ETV doses versus planned PTV doses.

## References

[1] Gregoire V., Jeraj R., Lee J.A., O’Sullivan B. (2012). Radiotherapy for head and neck tumors in 2012 and beyond: Conformal, tailored, and adaptive?. Lancet Oncol..

[2] Ahn P.H., Chen C.C., Ahn A.I., Hong L., Scripes P.G., Shen J., Lee C.-C., Miller E., Kalnicki S., Garget M.K. (2011). Adaptive planning in intensity-modulated radiotherapy for head and neck cancers: Single institution experience and clinical implications. Int. J. Radiat. Oncol. Biol. Phys..

[3] Bhide S.A., Davies M., Burke K., McNair H.A., Hansen V., Barbachano Y., El-Hariry I.A., Newbold K., Harrington K.J., Nutting C.M. (2010). Weekly volume and dosimetric changes during chemoradiotherapy with intensity-modulated radiotherapy for head and neck cancer: A prospective observational study. Int. J. Radiat. Oncol. Biol. Phys..

[4] Castelli J., Simon A., Rigaud B., Chajon E., Thariat J., Benezery K., Vauleon E., Jegoux F., Henry O., Lafond C. (2018). Adaptive radiotherapy in head and neck cancer is required to avoid tumor underdose. Acta Oncol..

[5] Chen C., Fei Z., Chen L., Bai P., Lin X., Pan J. (2014). Will weight loss cause significant dosimetric changes of target volumes and organs at risk in nasopharyngeal carcinoma treated with intensity-modulated radiotherapy?. Med. Dosim..

[6] Hansen E.K., Bucci M.K., Quivey J.M., Weinberg V., Xia P. (2006). Repeat CT imaging and replanning during the course of IMRT for head and neck cancer. Int. J. Radiat. Oncol. Biol..

[7] Schwartz D.L., Garden A.S., Shah S.J., Chronowski G., Seipal S., Rosenthal D.I., Chen Y., Zhang Y., Zhang L., Wong P.F. (2013). Adaptive radiotherapy for head and neck cancer—Dosimetric results from a prospective clinical trial. Radiother. Oncol..

[8] Wu Q., Chi Y., Chen P.Y., Krauss D.J., Yan D., Martinez A. (2009). Adaptive replanning strategies accounting for shrinkage in head and neck IMRT. Int. J. Radiat. Oncol. Bio. Phys..

[9] Yip C., Thomas C., Michaelidou A., James D., Lynn R., Lei M., Guerrero Urbano T. (2014). Co-registration of cone beam CT and planning CT in head and neck IMRT dose estimation: A feasible adaptive radiotherapy strategy. Br. J. Radiol..

[10] Zhang X., Li M., Cao J., Luo J.W., Xu G.Z., Gao L., Yi J., Huang X., Xiao J., Li S. (2012). Dosimetric variations of target volumes and organs at risk in nasopharyngeal carcinoma intensity modulated radiotherapy. Br. J. Radiol..

[11] Height R., Khoo V., Lawford C., Cox J., Joon D.L., Rolfo A., Wada M. (2010). The dosimetric consequences of anatomical changes in head and neck radiotherapy patients. J. Med. Imaging Radiat. Oncol..

[12] Marzi S., Pinnaro P., D’Alessio D., Strigari L., Bruzzaniti V., Giordano C., Giovinazzo G., Marucci L. (2012). Anatomical and dose changes of gross tumour volume and parotid glands for head and neck cancer patients during intensity-modulated radiotherapy: Effect on the probability of xerostomia incidence. Clin. Oncol..

[13] Van Beek S., van Kranen S., Mencarelli A., Remeijer P., Rasch C., van Herk M., Sonke J.J. (2010). First clinical experience with a multiple region of interest registration and correction method in radiotherapy of head and neck cancer patients. Radiother. Oncol..

[14] Van Kranen S., Mencarelli A., van Beek S., Rasch C., van Herk M., Sonke J.J. (2013). Adaptive radiotherapy with an average anatomy model: Evaluation and quantification of residual deformations in head and neck cancer patients. Radiother. Oncol..

[15] Rueckert D., Sonoda L.I., Hayes C., Hill D.L., Leach M.O., Hawkes D.J. (1999). Nonrigid registration using free-form deformations: Application to breast MR images. IEEE Trans. Med. Imaging.

[16] Mandrekar J. (2010). Receiver operating characteristic curve in diagnostic test assessment. Biostat. Clin. J. Thorac. Oncol..

[17] Olteanu L.A.M., Duprez F., De Neve W., Berwouts D., Vercauteren T., Bauters W., Deron P., Huvenne W., Bonte K., Goethals I. (2018). Late mucosal ulcers in dose-escalated adaptive dose-painting treatments for head and neck cancer. Acta Oncol..

[18] Yang H., Hu W., Wang W., Chen P., Ding W., Luo W. (2013). Replanning during intensity modulated radiation therapy improved quality of life in patients with nasopharyngeal carcinoma. Int. J. Radiat. Oncol. Biol. Phys..

[19] Chen A.M., Daly M.E., Cui J., Mathai M., Benedict S., Purdy J.A. (2014). Clinical outcomes among patients with head and neck cancer treated by intensity-modulated radiotherapy with and without replanning. Head Neck.

[20] Kataria T., Gupta D., Goyal S., Bisht S.S., Basu T., Abhishek A., Narang K., Banerjee S., Nasreen S., Sambasivamet S. (2016). Clinical outcomes of adaptive radiotherapy in head and neck cancers. Br. J. Radiol..

[21] Schwartz D.L., Garden A.S., Thomas J., Chen Y., Zhang Y., Lewin J., Chambers M.S., Dong L. (2012). Adaptive radiotherapy for head and neck cancer: Initial clinical outcomes from a prospective trial. Int. J. Radiat. Oncol. Biol. Phys..

[22] Brown E., Owen R., Harden F., Mengersen K., Oestreich K., Houghton W., Poulsen M., Harris S., Lin C., Porceddu S. (2015). Predicting the need for adaptive radiotherapy in head and neck cancer. Radiother. Oncol..

[23] Vickress J.R., Battista J., Barnett R., Yartsev S. (2018). Online daily assessment of dose change in head and neck radiotherapy without dose-recalculation. J. Appl. Clin. Med. Phys..

[24] Mencarelli A., van Kranen S.R., Hamming-Vrieze O., van Beek S., Rasch C.R., van Herk M., Sonke J.J. (2014). Deformable image registration for adaptive radiation therapy of head and neck cancer: Accuracy and precision in the presence of tumor changes. Int. J. Radiat. Oncol. Biol. Phys..

[25] Qin A., Lonascu D., Liang J., Han X., O’Connell N., Yan D. (2018). The evaluation of hybrid biomechanical deformable registration method on a multistage physical phantom with reproducible deformation. Radiat. Oncol..

[26] Zhong H., Chetty I.J. (2017). Caution must be exercised when performing deformable dose accumulation for tumors undergoing mass changes during fractionated radiation therapy. Int. J. Radiat. Oncol. Biol. Phys..

[27] Langendijk J.A., Lambin P., de Ruysscher D., Widder J., Bos M., Verheij M. (2013). Selection of patients for radiotherapy with protons aiming at reduction of side effects: The model based approach. Radiother. Oncol..

[28] Bibault J.E., Zapletal E., Rance B., Giraud P., Burgun A. (2018). Labeling for Big Data in radiation oncology: The Radiation Oncology Structures. PLoS ONE.

