# Supplementary material for: Deterioration of Intended Target Volume Radiation Dose Due to Anatomical Changes in Patients with Head-and-Neck Cancer
Source: Cancers (Basel). 2021 Aug 24;13(17):4253. doi: 10.3390/cancers13174253 (PMC8428222; doi:10.3390/cancers13174253)
Supplement: Supplementary file 1 [file cancers-13-04253-s001.zip › cancers-1298392-supplementary.pdf]

Supplementary material:

Supplementary Table S1: Details of patient positioning and planning CT, high and low dose clinical target volume delineation, dose prescription and planning objectives.

| | |
|---|---|
| Patient positioning and CT | Patients were immobilized with a 5-point thermoplastic mask and positioned with a knee support and head rest. Each patient had a contrast enhanced planning CT with 1 x 1 x $3mm^3$ voxel size (Somatom Sensation Open, Siemens AG, Erlangen, Germany). |
| Target volume delineation | The primary tumor and pathological nodes (GTV) were delineated based on clinical and imaging information available. A high risk CTV (CTV1) was created by expansion of GTV with 10 mm, allowing adjustment for anatomical borders. A low risk CTV (CTV2) was created around CTV1 by another expansion of 5 mm. Additionally, bilateral elective neck nodes were delineated and included in CTV2. A planning target volume (PTV) margin was constructed around all CTVs, 5 mm margin was used in patients treated before the 1st of April 2015 (90 patients), thereafter, a PTV margin of 3mm was applied (98 patients). |
| Dose prescription | The prescribed dose was 54.25 Gy in 35 fractions to low risk PTV (PTV2) and 70 Gy in 35 fractions to high risk PTV (PTV1), delivered using a simultaneous integrated boost technique. |
| Planning requirements | Planning objectives for optimization of PTVs were: dose to 99% of the volume ($D_{99}$) > 95% and $D_1$ < 107% of prescribed dose. Dose calculation in the first 4 mm from the external contour was considered unreliable and ignored for the target volume during plan optimization and analysis. |

Supplementary Table S2: Threshold violation of 1 and 2 Gy difference of accumulated versus planned dose

| | | ETV to PTV | | CTV to PTV | | PTV to PTV | | CTV to CTV | |
|---|---|---|---|---|---|---|---|---|---|
| | | 1 Gy | 2 Gy | 1 Gy | 2 Gy | 1 Gy | 2 Gy | 1 Gy | 2 Gy |
| Target 1 (*n* = 188) | $D_{99}$ | 15 (8%) | 4 (2%) | 3 (2%) | 2 (1%) | 79 (42%) | 39 (21%) | 26 (14%) | 8 (4%) |
| | $D_1$ | 7 (4%) | 4 (2%) | 9 (5%) | 4 (2%) | 7 (4%) | 3(2%) | 6 (3%) | 4 (2%) |
| | | ETV to PTV | | CTV to PTV | | PTV to PTV | | CTV to CTV | |
| | | 1 Gy | 2 Gy | 1 Gy | 2 Gy | 1 Gy | 2 Gy | 1 Gy | 2 Gy |
| Target 2 (*n* = 177) | $D_{99}$ | 23 (13%) | 7 (4%) | 3 (2%) | 1 (1%) | 56 (32%) | 14 (8%) | 15 (8%) | 5 (3%) |
| | $D_1$ | 7 (4%) | 2 (1%) | 10 (6%) | 2 (1%) | 5 (3%) | 2 (1%) | 5 (3%) | 2 (1%) |

Supplementary Table S3a: Association of characteristics with deterioration over 1 and 2 Gy of the accumulated $D_{99}$_ETV1 dose versus the planned $D_{99}$_PTV1 dose.

| **Target Volume 1** | **$D_{99}$ (Threshold 1 Gy Deterioration)** | | | **$D_{99}$ (Threshold 2 Gy Deterioration)** | | |
|---|---|---|---|---|---|---|
| N | <1 Gy: 173 | >1 Gy: 15 | *p* | <2 Gy:184 | >2 Gy:4 | *P* |
| Age (years, (SD)) | 61.0 (10.5) | 62.2 (14.4) | 0.681 | 61.0 (10.9) | 64.0 (5.5) | 0.588 |
| Gender (*n*, (%)) | | | | | | |
| Male | 121 (70) | 12 (80) | | 131 (71) | 2 (40) | |
| Female | 52 (30) | 3 (20) | 0.559 | 53 (29) | 3 (60) | 0.582 |
| Weight (kg, (SD)) | 76.1 (71.6) | 77.5 (22.0) | 0.774 | 76.3 (17.7) | 74.5 (30.1) | 0.843 |
| BMI (kg/m$^2$,(SD)) | 24.9 (5.2) | 24.9 (8.0) | 0.958 | 24.8 (5.3) | 26.2 (13.4) | 0.852 |
| Location (*n*, (%)) | | | | | | |
| Nasopharynx | 30 (17) | 1 (7) | | 31 (17) | 0 (0) | |
| Oroph/oral cavity | 79 (48) | 7 (47) | | 83 (45) | 3 (75) | |
| Larynx/Hypoph | 38 (22) | 5 (33) | | 42 (23) | 1 (25) | |
| Other | 26 (15) | 2 (13) | 0.623 | 28 (15) | 0 (0) | 0.561 |
| Pathology (*n*, (%)) | | | | | | |
| Squamous cell | 159 (92) | 15 (100) | | 170 (92) | 4 (100) | |
| Other | 14 (8) | 0 (0) | 0.608 | 14 (8) | 0 (0) | 1.000 |
| T stage (*n*, (%)) | | | | | | |
| T0/T1/T2 | 102 (59) | 7 (47) | | 108 (59) | 1 (25) | |
| T3/T4 | 71 (41) | 8 (53) | 0.355 | 76 (41) | 3 (75) | 0.311 |
| N stage (*n*, (%)) | | | | | | |
| N0 | 60 (35) | 6 (40) | | 63 (34) | 3 (75) | |
| N1-3 | 113 (65) | 9 (60) | 0.679 | 121 (66) | 1 (25) | 0.125 |
| N side (*n*, (%)) | | | | | | |
| Unilateral | 60 (53) | 6 (67) | | 66 (54) | 0 (0) | |
| Bilateral | 53 (47) | 3 (33) | 0.505 | 55 (46) | 1 (100) | 0.459 |
| N largest size (cm, (SD)) | 2.3 (1.6) | 2.4 (1.5) | 0.933 | 2.4 (1.6) | 1.1 (0) | 0.439 |
| N largest size (*n*, (%)) | | | | | | |
| <3 cm | 77 (68) | 5 (56) | | 81 (67) | 1 (100) | |
| >3 cm | 36 (32) | 4 (44) | 0.473 | 40 (33) | 0 (0) | 1.000 |
| Dahanca (*n*, (%)) | | | | | | |
| No | 88 (51) | 6 (40) | | 93 (50) | 1 (25) | |
| Yes | 85 (49) | 9 (60) | 0.419 | 91 (50) | 3 (75) | 0.621 |
| Chemotherapy (*n*, (%)) | | | | | | |
| No | 86 (50) | 10 (67) | | 94 (51) | 2 (50) | |
| Yes | 87 (50) | 5 (33) | 0.208 | 90 (49) | 2 (50) | 1.000 |
| PTV margin (*n*, (%)) | | | | | | |
| 5 mm | 90 (52) | 0 (0) | | 90 (49) | 0 (0) | |
| 3 mm | 83 (48) | 15 (100) | **<0.0001** | 94 (51) | 4 (100) | 0.122 |

Supplementary Table S3b: Association of characteristics with deterioration over 1 and 2 Gy of the accumulated $D_{99}$_ETV2 dose versus the planned $D_{99}$_PTV2 dose.

| **Target Volume 2** | **D99 (Threshold 1 Gy Deterioration)** | | | **D99 (Threshold 2 Gy Deterioration)** | | |
|---|---|---|---|---|---|---|
| N | <1 Gy: 154 | >1 Gy: 23 | *p* | <2 Gy:170 | >2 Gy:7 | *p* |
| Age (years, (SD)) | 60.6 (10.7) | 62.5 (12.9) | 0.427 | 61.0 (11.1) | 58.4 (8.5) | 0.558 |
| Gender (*n*, (%)) | | | | | | |
| Male | 107 (70) | 19 (83) | | 123 (72) | 3 (43) | |
| Female | 47 (30) | 4 (17) | 0.227 | 47 (28) | 4 (57) | 0.107 |
| Weight (kg, (SD)) | 74.8 (17.3) | 86.5 (20.0) | **0.003** | 75.7 (17.9) | 89.7 (27.3) | 0.227 |
| BMI ($kg/m^2$,(SD)) | 24.4 (5.2) | 27.7 (7.0) | **0.007** | 24.6 (5.1) | 31.0 (10.5) | 0.160 |
| Location (*n*, (%)) | | | | | | |
| Nasopharynx | 27 (17) | 2 (9) | | 28 (17) | 1 (14) | |
| Oroph/oral cavity | 70 (46) | 12 (52) | | 78 (46) | 4 (57) | |
| Larynx/Hypoph | 36 (23) | 6 (26) | | 40 (23) | 2 (29) | |
| Other | 21 (14) | 3 (13) | 0.749 | 24 (14) | 0 (0) | 0.738 |
| Pathology (*n*, (%)) | | | | | | |
| Squamous cell | 144 (94) | 22 (96) | | 159 (94) | 7 (100) | |
| Other | 10 (6) | 1 (4) | 0.691 | 11 (6) | 0 (0) | 1.000 |
| T stage (*n*, (%)) | | | | | | |
| T0/T1/T2 | 90 (58) | 12 (52) | | 99 (58) | 3 (43) | |
| T3/T4 | 64 (42) | 11 (48) | 0.570 | 71 (42) | 4 (57) | 0.459 |
| N stage (*n*, (%)) | | | | | | |
| N0 | 51 (33) | 7 (30) | | 55 (32) | 3 (43) | |
| N1-3 | 103 (67) | 16 (70) | 0.798 | 115 (68) | 4 (57) | 0.685 |
| N side (*n*, (%)) | | | | | | |
| Unilateral | 54 (52) | 9 (56) | | 62 (54) | 1 (25) | |
| Bilateral | 49 (48) | 7 (44) | 0.776 | 53 (46) | 3 (75) | 0.341 |
| N largest size (cm, (SD)) | 2.3 (1.6) | 2.4 (1.5) | 0.921 | 2.3 (1.6) | 2.4 (1.8) | 0.988 |
| N largest size (*n*, (%)) | | | | | | |
| <3 cm | 69 (67) | 10 (63) | | 77 (67) | 2 (50) | |
| >3 cm | 34 (33) | 6 (38) | 0.724 | 38 (33) | 2 (50) | 0.602 |
| Dahanca (*n*, (%)) | | | | | | |
| No | 75 (49) | 10 (44) | | 80 (47) | 5 (71) | |
| Yes | 79 (51) | 13 (57) | 0.640 | 90 (53) | 2 (29) | 0.263 |
| Chemotherapy (*n*, (%)) | | | | | | |
| No | 73 (47) | 13 (57) | | 83 (49) | 3 (43) | |
| Yes | 81 (53) | 10 (43) | 0.414 | 87 (51) | 4 (57) | 1.000 |
| PTV margin (*n*, (%)) | | | | | | |
| 5 mm | 81 (53) | 3 (13) | | 84 (49) | 0 (0) | |
| 3 mm | 73 (47) | 20 (87) | **0.001** | 86 (51) | 7 (100) | 0.015 |

Supplementary Table S3c: Association of characteristics with deterioration over 1 and 2 Gy of the accumulated $D_1$_ETV1 dose versus the planned $D_1$_PTV1 dose.

| **Target Volume 1** | **D1 (Threshold 1 Gy Deterioration)** | | | **D1 (Threshold 2 Gy Deterioration)** | | |
|---|---|---|---|---|---|---|
| N | <1 Gy: 181 | >1 Gy: 7 | *p* | <2 Gy:184 | >2 Gy:4 | *p* |
| Age (years, (SD)) | 60.9 (10.9) | 66.9 (4.5) | **0.011** | 61.0 (10.9) | 66.3 (5.7) | 0.337 |
| Gender (*n*, (%)) | | | | | | |
| Male | 127 (70) | 6 (86) | | 129 (70) | 4 (100) | |
| Female | 54 (30) | 1 (14) | 0.676 | 55 309) | 0 (0) | 0.323 |
| Weight (kg, (SD)) | 75.6 (17.7) | 90.6 (16.9) | **0.030** | 76.0 (17.9) | 86.4 (15.9) | 0.254 |
| BMI (kg/m$^2$,(SD)) | 24.7 (5.0) | 28.9 (8.3) | **0.048** | 24.8 (5.5) | 25.4 (5.1) | 0.846 |
| Location (*n*, (%)) | | | | | | |
| Nasopharynx | 29 (16) | 2 (29) | | 31 (17) | 0 (0) | |
| Oroph/oral cavity | 85 (47) | 1 (14) | | 85 (46) | 1 (25) | |
| Larynx/Hypoph | 39 (22) | 4 (57) | | 40 (22) | 3 (75) | |
| Other | 28 (15) | 0 (0) | 0.071 | 28 (15) | 0 (0) | 0.089 |
| Pathology (*n*, (%)) | | | | | | |
| Squamous cell | 167 (92) | 7 (100) | | 170 (92) | 4 (100) | |
| Other | 14 (8) | 0 (0) | 1.000 | 14 (8) | 0 (0) | 1.000 |
| T stage (*n*, (%)) | | | | | | |
| T0/T1/T2 | 108 (60) | 1 (14) | | 108 (59) | 1 (25) | |
| T3/T4 | 73 (40) | 6 (86) | **0.043** | 76 (41) | 3 (75) | 0.311 |
| N stage (*n*, (%)) | | | | | | |
| N0 | 63 (35) | 3 (43) | | 65 (35) | 1 (25) | |
| N1-3 | 118 (65) | 4 (57) | 0.698 | 119 (65) | 3 (75) | 1.000 |
| N side (*n*, (%)) | | | | | | |
| Unilateral | 64 (54) | 2 (50) | | 64 (54) | 2 (67) | |
| Bilateral | 54 (46) | 2 (50) | 1.000 | 55 (46) | 1 (33) | 1.000 |
| N largest size (cm, (SD)) | 2.3 (1.6) | 1.5 (1.0) | 0.289 | 2.4 (1.6) | 1.0 (2.0) | 0.143 |
| N largest size (*n*, (%)) | | | | | | |
| <3 cm | 79 (67) | 3 (75) | | 79 (66) | 3 (100) | |
| >3 cm | 39 (33) | 1 (25) | 0.473 | 40 (34) | 0 (0) | 0.550 |
| Dahanca (*n*, (%)) | | | | | | |
| No | 90 (50) | 4 (57) | | 91 (50) | 3 (75) | |
| Yes | 91 (50) | 3 (43) | 1.000 | 93 (50) | 1 (25) | 0.621 |
| Chemotherapy (*n*, (%)) | | | | | | |
| No | 93 (51) | 3 (43) | | 93 (51) | 3 (75) | |
| Yes | 88 (49) | 4 (57) | 0.716 | 91 (49) | 1 (25) | 0.621 |
| PTV margin (*n*, (%)) | | | | | | |
| 5 mm | 86 (48) | 3 (43) | | 89 (48) | 1 (25) | |
| 3 mm | 95 (53) | 4 (57) | 0.711 | 95 (52) | 3 (75) | 0.622 |

Supplementary Table S3d: Association of characteristics with deterioration over 1 and 2 Gy of the accumulated $D_1$_ETV2 dose versus the planned $D_1$_PTV2 dose.

| **Target Volume 2** | **D1 (Threshold 1 Gy Deterioration)** | | | **D1 (Threshold 2 Gy Deterioration)** | | |
|---|---|---|---|---|---|---|
| N | <1 Gy: 170 | >1 Gy: 7 | *p* | <2 Gy: 175 | >2 Gy: 2 | *p* |
| Age (years, (SD)) | 60.6 (11.1) | 66.8 (4.5) | **0.009** | 60.8 (11.0) | 62.5 (5.0) | 0.828 |
| Gender (*n*, (%)) | | | | | | |
| Male | 120 (71) | 6 (86) | | 124 (71) | 2 (100) | |
| Female | 50 (29) | 1 (14) | 0.675 | 51 (29) | 0 (0) | 1.000 |
| Weight (kg, (SD)) | 75.7 (17.9) | 90.6 (16.9) | **0.032** | 76.1 (18.0) | 94.0 (19.8) | 0.164 |
| BMI (kg/m$^2$,(SD)) | 24.7 (5.4) | 28.9 (8.3) | **0.048** | 24.8 (5.6) | 27.6 (3.3) | 0.480 |
| Location (*n*, (%)) | | | | | | |
| Nasopharynx | 27 (16) | 2 (29) | | 29 (17) | 0 (0) | |
| Oroph/oral cavity | 81 (48) | 1 (14) | | 82 (47) | 0 (0) | |
| Larynx/Hypoph | 38 (22) | 4 (57) | | 40 (23) | 2 (100) | |
| Other | 24 (14) | 0 (0) | 0.082 | 24 (14) | 0 (0) | 0.090 |
| Pathology (*n*, (%)) | | | | | | |
| Squamous cell | 159 (94) | 7 (100) | | 164 (94) | 2 (100) | |
| Other | 11 (6) | 0 (0) | 0.487 | 11 (6) | 0 (0) | 1.000 |
| T stage (*n*, (%)) | | | | | | |
| T0/T1/T2 | 101 (59) | 1 (14) | | 102 (58) | 0 (0) | |
| T3/T4 | 69 (41) | 6 (86) | **0.043** | 73 (42) | 2 (100) | 0.178 |
| N stage (*n*, (%)) | | | | | | |
| N0 | 51 (33) | 7 (30) | | 58 (33) | 0 (0) | |
| N1-3 | 103 (67) | 16 (70) | 0.798 | 117 (67) | 2 (100) | 1.000 |
| N side (*n*, (%)) | | | | | | |
| Unilateral | 54 (52) | 9 (56) | | 62 (54) | 1 (25) | |
| Bilateral | 49 (48) | 7 (44) | 0.776 | 53 (46) | 3 (75) | 1.000 |
| N largest size (cm, (SD)) | 2.4 (1.6) | 1.5 (1.0) | 0.291 | 2.6 (1.6) | 1.1 (1.4) | 0.274 |
| N largest size (*n*, (%)) | | | | | | |
| <3 cm | 76 (66) | 3 (75) | | 77 (66) | 2 (100) | |
| >3 cm | 39 (34) | 1 (25) | 1.000 | 40 (34) | 0 (0) | 0.550 |
| Dahanca (*n*, (%)) | | | | | | |
| No | 81 (48) | 4 (57) | | 84 (48) | 1 (50) | |
| Yes | 89 (52) | 3 (43) | 0.712 | 91 (52) | 1 (50) | 1.000 |
| Chemotherapy (*n*, (%)) | | | | | | |
| No | 83 (49) | 3 (43) | | 85 (49) | 1 (50) | |
| Yes | 87 (51) | 4 (57) | 1.000 | 90 (51) | 1 (50) | 1.000 |
| PTV margin (*n*, (%)) | | | | | | |
| 5 mm | 90 (53) | 3 (43) | | 92 (53) | 1 (50) | |
| 3 mm | 80 (47) | 4 (57) | 0.709 | 83 (47) | 1 (50) | 1.000 |

Supplementary Table S4: P values of the independed sample t-test evaluating differences between patients with and without dosimetric deterioration (delivered ETV doses versus planned PTV doses in TV1 and TV2 with tresholds of 1 and 2Gy dose deterioration), testing weight change and tube feeding during treatment.

| | D99 | | D99 | | D1 | | D1 | |
|---|---|---|---|---|---|---|---|---|
| | TV1(1 Gy) | TV1(2 Gy) | TV2(1 Gy) | TV2(2 Gy) | TV1(1 Gy) | TV1(2 Gy) | TV2(1 Gy) | TV2(2 Gy) |
| ΔG_w1 | 0.151 | **0.007** | 0.685 | 0.947 | 0.764 | 0.630 | 0.702 | 0.286 |
| ΔG_w2 | **0.000** | **0.023** | 0.367 | 0.904 | 0.733 | 0.294 | 0.742 | 0.746 |
| ΔG_w3 | **0.006** | 0.242 | 0.732 | 0.986 | 0.697 | 0.262 | 0.707 | 0.844 |
| ΔG_w4 | **0.042** | 0.617 | **0.046** | **0.044** | 0.192 | 0.095 | 0.210 | **0.004** |
| ΔG_w5 | 0.082 | 0.550 | 0.099 | 0.071 | 0.262 | 0.071 | 0.289 | **0.004** |
| ΔG_w6 | 0.097 | 0.551 | 0.816 | 0.863 | 0.304 | 0.181 | 0.328 | **0.010** |
| Tube feeding | 0.952 | 0.115 | 0.614 | 0.694 | 0.691 | 1.000 | 0.694 | 1.000 |

Supplementary Table S5: Association of clinical ART with dosimetric deterioration over 1 and 2Gy of accumulated ETV doses versus planned PTV doses, results of the Chi-Square-tests (or Fisher's exact-test in case of less than 5 events in at least one category) evaluating differences between patients with and without dosimetric deterioration.

| | 1 Gy Threshold | | | 2 Gy Threshold | | |
|---|---|---|---|---|---|---|
| Clinical ART | TV1 $D_{99}$ (*n*, (%)) | | | TV1 $D_{99}$ (*n*, (%)) | | |
| | <1 Gy: *n* = 173 | >1 Gy: *n* = 15 | *p* | <2 Gy: *n* = 184 | >2 Gy: *n* = 4 | *p* |
| No | 154 (89) | 10 (67) | | 162 (88) | 2 (50) | |
| Yes | 19 (11) | 5 (33) | **0.013** | 22 (12) | 2 (50) | 0.080 |
| | TV2 $D_{99}$ (*n*, (%)) | | | TV2 $D_{99}$ (*n*, (%)) | | |
| | <1 Gy: *n* = 154 | >1 Gy: *n* = 23 | *p* | <2 Gy: *n* = 170 | >2 Gy: *n* = 7 | *p* |
| No | 134 (87) | 19 (83) | | 147 (87) | 6 (86) | |
| Yes | 20 (13) | 4 (17) | 0.523 | 23 (13) | 1 (14) | 1.000 |
| | TV1 $D_1$ (*n*, (%)) | | | TV1 $D_1$ (*n*, (%)) | | |
| | <1 Gy: *n* = 181 | >1 Gy: *n* = 7 | p | <2 Gy: *n* = 184 | >2 Gy: *n* = 4 | *p* |
| No | 158 (87) | 6 (86) | | 161 (88) | 3 (75) | |
| Yes | 23 (13) | 1 (14) | 0.902 | 23 (12) | 1 (25) | 0.424 |
| | TV2 $D_1$ (n, (%)) | | | TV2 $D_1$ (n, (%)) | | |
| | <1Gy: n=170 | >1Gy: n=7 | p | <2Gy: n=175 | >2Gy: n=2 | p |
| No | 147 (87) | 6 (86) | | 152 (87) | 1 (50) | |
| Yes | 23 (13) | 1 (14) | 1.000 | 23 (13) | 1 (50) | 0.253 |
